# The impact of machine learning on ethological neuroscience

**DOI:** 10.3389/fnbeh.2025.1745658

**Published:** 2026-01-09

**Authors:** Guillermo Hidalgo-Gadea, Onur Güntürkün, Mehdi Behroozi

**Affiliations:** 1 Department of Biopsychology, Faculty of Psychology, Ruhr University Bochum, Bochum, Germany; 2 Research Center One Health Ruhr University Alliance Ruhr, Faculty of Psychology, Ruhr University Bochum, Bochum, Germany; 3 Cognitive Neurobiology, Research Center One Health Ruhr University Alliance Ruhr, Faculty of Biology and Biotechnology, Ruhr University Bochum, Bochum, Germany

**Keywords:** animal behavior, ethology, machine learning, naturalistic behavior, neuroscience, pose estimation

## Abstract

Machine learning is revolutionizing behavioral neuroscience by enabling the study of animal behavior with greater ecological validity while maintaining experimental rigor. Traditional manual observation methods in ethology are constrained by subjectivity, costs, and low throughput, whereas modern machine learning algorithms now provide quantitative tools to investigate natural behavior with unprecedented precision. This mini review surveys recent advances in machine learning for behavioral neuroscience, focusing on markerless pose estimation and unsupervised behavioral clustering, and discusses their roles along the typical research pipeline, from tracking and detection to classification and integration of behavioral and neural data. Open-source platforms using deep learning–based image processing have turned video cameras into high-resolution measurement devices, while unsupervised methods extend inference across large-scale behavioral recordings. In laboratory settings, machine learning enables fine-scale analysis of animal kinematics and their relationship to neural activity, while in field studies it enhances longitudinal data collection through drone and satellite imaging. These approaches expand ethological research by quantifying movement, segmenting behavior into meaningful units, detecting transient events often missed by human observers, and bridging behavior with brain activity via joint latent spaces and closed-loop paradigms. Although challenges remain in handling high-dimensional datasets, machine learning offers powerful opportunities for more comprehensive neuroscientific insights. By bridging the controlled precision of the laboratory with the complexity of real-world environments, these methods advance our understanding of animal behavior and its neural underpinnings, providing experimentalists with practical tools to design, implement, and interpret more naturalistic studies in the field of ethological neuroscience.

## 1 Introduction

Hirsch (1986) famously remarked that “Nothing in neurobiology makes sense–except in the light of behavior.” Indeed, understanding behavior is fundamental to neuroscience, yet it has long presented a methodological bottleneck. Unlike neural signals, which can be precisely captured with modern recording technologies, behavior unfolds continuously across multiple spatial and temporal scales and is notoriously difficult to measure objectively (Pereira et al., 2020; Calhoun and El Hady, 2023). Ethological neuroscience emerged to counteract the limitations of highly controlled laboratory paradigms, which often minimize behavior variability at the expense of ecological validity. By prioritizing spontaneous and naturalistic responses, it brings experimental conditions closer to the ecological context in which neural circuits evolved, without overreliance on learned task performance. This approach demonstrates the engagement of only a fraction of the neural repertoire at stereotyped laboratory tasks that evolved for complex and naturalistic behaviors. However, this ecological focus places even greater demands on behavior quantification (Gomez-Marin et al., 2014; Krakauer et al., 2017). Early methods, whether hand-coded ethograms or task-specific scoring systems, were labor-intensive, subjective, and often restricted to simplified paradigms, which even require weeks to manually analyze hours of video to track only a handful of predetermined behaviors. These constraints left a persistent gap between the richness of behavior in natural settings and the precision of laboratory-based measurements (Gomez-Marin et al., 2014; Sejnowski et al., 2014).

Machine learning (ML) emerged as a solution to bridge this gap. From its statistical roots in regression and classification, ML evolved into deep architectures such as convolutional and recurrent neural networks that can extract complex patterns from high-dimensional data (Krizhevsky et al., 2012). These advances in computer vision and pattern recognition opened the door to automated analysis of complex biological data (Pichler and Hartig, 2023). Among neuroscience applications, behavioral research has seen the most transformative impact: video-based ML now enables scalable, non-invasive quantification of spontaneous movement with unprecedented precision. What once required weeks of manual scoring can now be processed automatically in hours, turning ordinary video into structured datasets that capture naturalistic behavior across laboratory and ecological contexts (Pereira et al., 2020; Couzin and Heins, 2023).

This mini review outlines how ML is reshaping ethological neuroscience by enabling high-resolution, unbiased, and scalable quantification of natural behaviors, linking them to neural activity in ways that were previously impossible. We focus specifically on video-based methods, as they have seen the most rapid adoption and the greatest impact on experimental practice. The next sections are organized along a pipeline that mirrors the research process: the tracking of animals in space and time (Section “2.1 Tracking animals in space and time”), the detection and classification of actions (Section “2.2 Detecting actions and classifying behavior”), and the integration of behavioral data with neural recordings and internal states (Section “2.3 Linking behavior with neural activity”). For each domain, we highlight the conceptual advances introduced by ML and situate widely used tools within this framework. For a comprehensive overview, see Mathis and Mathis (2020), Couzin and Heins (2023), Luxem et al. (2023), Nagy et al. (2023), Saoud et al. (2024), Vogg et al. (2025). Together, these developments illustrate how ML enables a synthesis of laboratory precision and ecological validity, offering new opportunities to study the neural basis of natural behavior.

## 2 Main

### 2.1 Tracking animals in space and time

One of the challenges in ethological neuroscience is obtaining precise, objective, and high-throughput measures of animal movement. Traditional methods like scoring sheets were labor-intensive, subjective, and constrained in resolution, limiting the scale of behavioral experiments. ML, and in particular convolutional neural networks (CNNs), has revolutionized this field with automated video analysis across diverse contexts. By turning ordinary video cameras into high-resolution tools, these approaches bridge the gap between ecological validity and experimental rigor.

Before ML, computer vision relied on simple techniques like edge detection or blob tracking, which captured only coarse movement and often fails in complex environments. CNNs shifted this paradigm by extracting high-level visual features, allowing detection, classification, and tracking animals across varied setting. From specialized laboratory cameras to consumer devices, drones, CCTV, and camera traps, they enable studying freely moving animals in controlled and naturalistic environments. This revolutionized behavioral research, turning raw video into structured, quantitative datasets.

Tracking levels vary by research requirements. Broadly, image classification, object detection and centroid tracking focus on identifying the animal’s presence and location. YOLO (Redmon et al., 2016), a real-time object detection algorithm excels in fast tracking over many frames, prioritizing location over orientation or pose. More advanced tracking systems often use such object detection to crop complex scenes before applying additional tracking techniques to the isolated regions of interest. This top-down approach is particularly effective in multi-animal tracking systems like maDLC (Lauer et al., 2022). Other multi-animal tracking tools like TRex (Walter and Couzin, 2021) and idtracker.ai (Romero-Ferrero et al., 2019) integrate computer vision and CNNs to handle identification in crowded scenes. These approaches are particularly useful for collective behavior or field studies, where orientation or fine-scale kinematics may be less critical than monitoring overlapping trajectories across time and space.

For many ethological questions, however, it is essential to move beyond location and capture markerless, keypoint-based pose tracking. CNNs can be trained to identify specific body parts (e.g., nose, ears, limbs) to form a keypoint-based skeletal representation of the animal, enabling quantification of posture and kinematics such as joint angles. Widely adopted tools such as DeepLabCut (Nath et al., 2019), SLEAP (Pereira et al., 2022), MARS (Segalin et al., 2021), and DeepPoseKit (Graving et al., 2019) have made markerless tracking highly flexible across species and behaviors (see Monsees et al., 2022; Moore et al., 2022; Kirkpatrick et al., 2022 for x-ray video tracking examples), establishing it as a cornerstone of non-invasive, naturalistic neuroscience. For instance, in wild primates the use of pose tracking has been used to quantify facial resemblance of mandrills, revealing a paternal kin signal in social affiliations (Charpentier et al., 2020), or to track multi-individual movements of wild chimpanzees and bonobos in complex forest habitats, enabling fine-scale measurement of locomotor and social behavior in the field (Wiltshire et al., 2023). Similarly, audio and video tracking has been used to identify nut-cracking and drumming behavior in wild chimpanzees, uncovering age- and sex-specific patterns in these communicative and foraging behaviors (Bain et al., 2019).

More recently, 3D pose estimation has expanded what can be measured from video beyond 2D tracking. Multi-view triangulation methods reconstruct 3D trajectories by combining synchronized camera feeds (Nourizonoz et al., 2020; Sheshadri et al., 2020; Karashchuk et al., 2021; Ebrahimi et al., 2023), while 3D lifting approaches infer spatial structure directly from single-camera recordings. These models learn geometric constraints from paired 2D–3D data and exploit the fact that animal movement occupies only a limited subset of possible postures (Gosztolai et al., 2021). More recent volumetric CNNs integrate multiple camera views into 3D voxel representations rather than triangulated coordinates, allowing the network to learn spatial geometry directly and avoid reprojection errors (Abbaspoor et al., 2023). Related architectures also integrate temporal information directly within convolutional space (Grinciunaite et al., 2016; Reddy et al., 2021). Although still developing, these methods capture the spatial and temporal richness of animal interactions with unprecedented precision, providing a foundation for truly three-dimensional ethological analyses. For example, 3D tracking has been used to quantify how spatial and temporal movement patterns evolve during learning in freely moving macaques, revealing systematic changes in object interaction sequences as task proficiency increases (Abbaspoor et al., 2023). Similarly, 3D reconstruction of tool-tip trajectories in carrion crows showed how motor precision and movement stereotypy emerge with practice, providing fine-grained insight into the development of skilled tool use in birds (Moll et al., 2025).

A complementary strategy leverages marker-based motion capture to improve model training. Systems such as DANNCE (Dunn et al., 2021) and 3D-POP (Naik et al., 2023) integrate motion capture coordinates into CNN training pipelines, substantially improving tracking accuracy with significantly less manual labeling. These models generalize to markerless recordings, enabling accurate pose estimation where markers are impractical. Additionally, with overtrained models on large datasets, these systems can predict accurate 3D poses from single-view videos by leveraging the learned geometry of the marker-based skeleton.

**FIGURE 1:**
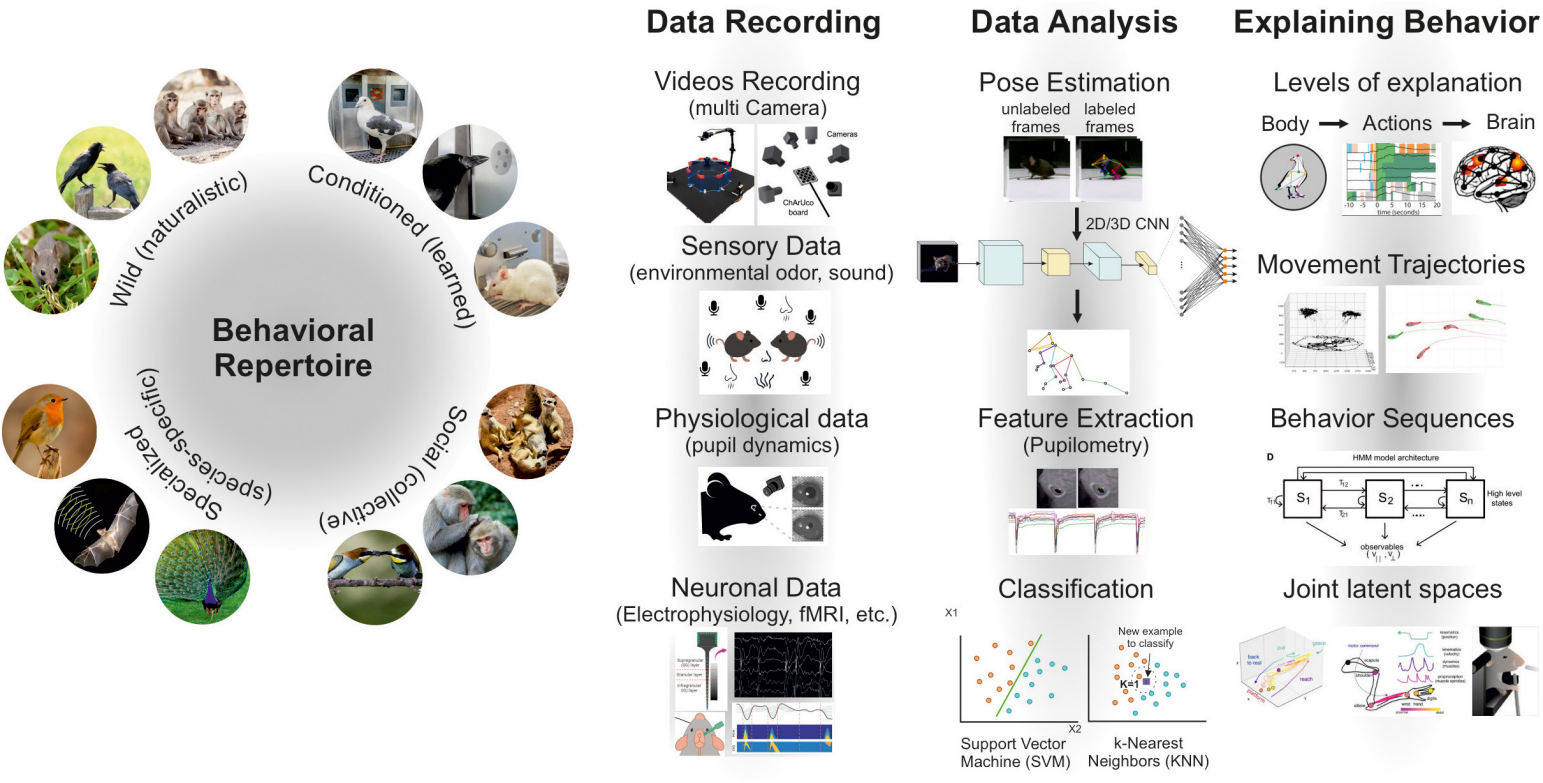
Integrative framework for ethological neuroscience using machine learning. This schematic summarizes stages for quantification and interpretation of animal behavior with contemporary computational tools. Left: behavioral repertoires range from naturalistic and species-typical actions to conditioned and socially coordinated behaviors, illustrating ecological and cognitive diversity across animal models. Center: multimodal data recording integrates multi-camera video capture, sensory environment monitoring (e.g., sound, odor), physiological measures (e.g., pupil dynamics) and neuronal recordings (electrophysiology, imaging). Middle–right: data analysis pipelines apply pose estimation and feature extraction to video and physiological signals, followed by classifiers and pattern-discovery algorithms (e.g., SVM, k-NN, CNNs) to identify behavioral units and trajectories. Right: the final column explicitly links analysis outputs to explanatory levels, from body kinematics, through actions and movement trajectories, to neural activity. Showing how machine learning uncovers behavioral sequences, hierarchical structure, and joint latent spaces that bridge behavior and brain activity.

Together, these tracking methods represent a profound shift in the structure of behavioral datasets. They transform raw video into continuous, high-dimensional time series of body-part coordinates, forming the basis for reproducible, shareable datasets across species and laboratories (Ye et al., 2024). This open-source ecosystem allows researchers to build on pretrained models and existing data, reducing redundancy and advancing comparative work. By converting raw video into structured, quantitative behaviors, CNN-based tracking now underpins modern ethological neuroscience, revealing subtle behavioral motifs, multi-individual interactions, and long-term dynamics that often escape human observation (Figure 1).

### 2.2 Detecting actions and classifying behavior

While pose tracking provides kinematic data, it must be translated to behavioral categories. Ethological neuroscience seeks not only to capture how animals move, but also to understand what they do, its timing, and sequence. Manual annotation is slow, subjective, and limits throughput and discovery. ML methods address these issues by automatically segmenting high-dimensional movement data into discrete behavioral motifs and uncovering both sub-second and large-scale patterns.

Behavior classification roughly splits into supervised and unsupervised methods. Supervised approaches rely on labeled training data, ideal for specific hypotheses about predefined behaviors. Classical algorithms such as k-nearest neighbors, support vector machines, or random forests have been successfully applied to learn feature representations from pose data and classify behavior with high accuracy. For instance, supervised classification methods have been used to detect *Drosophila* behaviors specific to aggression and courtship (Dankert et al., 2009), to track the behavioral repertoire of pigeons (Wittek et al., 2023), or to analyze mice behavior, revealing precise patterns of grooming, rearing, and locomotion, and enabling the quantification of behavioral differences linked to genetic or pharmacological manipulations (Kabra et al., 2013).

In contrast, unsupervised methods identify patterns without pre-labeled examples, enabling a more exploratory perspective. They use dimensionality reduction and sequence modeling to embed pose data into latent spaces, from which statistically distinct behavioral motifs can be extracted. For example, VAME (Luxem et al., 2022) and B-SOiD (Hsu and Yttri, 2021) apply deep-learning and clustering algorithms to identify recurring motifs, while keypoint-MoSeq (Weinreb et al., 2024) employs probabilistic sequence modeling to capture temporal dynamics and recurring action patterns. Behavioral Flow Analysis (von Ziegler et al., 2024) extends this approach by linking behavior quantification to statistical testing, summarizing behavior via the transition structure between stabilized clusters. This low-dimensional fingerprinting increases statistical power and improves cross-experiment comparability.

Benchmarks show that different unsupervised methods produce distinct behavioral segmentations, emphasizing the importance of method choice (Mlost et al., 2025). Based on this comparison, B-SOiD excels at fine-grained kinematic clustering, VAME captures smooth latent dynamics, Keypoint-MoSeq identifies temporally structured motifs, and Behavioral Flow Analysis is particularly suited for cross-dataset comparability and group-level statistical testing. However, supervised methods retain the advantage that performance can be readily evaluated against manual labels, facilitating quantification of accuracy and reliability.

By translating movement into meaningful behavioral units, ML enables ethological neuroscience to uncover structure in what once appeared as continuous variability. Increasingly, hybrid approaches that combine supervised and unsupervised strategies are emerging to integrate hypothesis-driven testing with exploratory discovery.

### 2.3 Linking behavior with neural activity

Understanding how neural activity gives rise to behavior has long been limited by a mismatch in resolution: neural recordings offer millisecond precision, whereas behavioral measurements were often coarse and categorical. ML-based tracking is closing this gap by providing automated, high-resolution measures of movement and posture that can be directly aligned with neural data. This convergence enables more naturalistic studies where brain activity and internal states are linked to behavioral dynamics.

Machine learning methods are increasingly used to uncover structure in large-scale neural recordings and their relationship to behavior. Dimensionality reduction techniques such as PCA and UMAP condense high-dimensional activity into interpretable, lower-dimensional components (Cunningham and Yu, 2014). Supervised deep-learning approaches can decode sensory inputs or predict motor intentions from neural data (Yamins and DiCarlo, 2016), while recurrent neural networks (RNNs) capture temporal dynamics of decision-making and motor control (Mante et al., 2013). Yet, these approaches traditionally treated neural data in isolation. Recent work shows that spontaneous movement explains a large share of cortical variability, emphasizing the need for behaviorally grounded analyses (Musall et al., 2019).

New methods now combine behavioral and neural data into shared latent spaces. Generalized linear model–hidden Markov models describe how transitions between behavioral states relate to shifts in neural activity (Calhoun et al., 2019), identifying behavioral state transitions in freely moving mice that correspond to shifts in cortical population activity, linking spontaneous actions with internal neural dynamics. Similarly, contrastive and self-supervised learning approaches such as CEBRA (Schneider et al., 2023) identify subtle correspondences between brain activity and ongoing behavior, revealing place- and head-direction–tuned neurons in the rat hippocampus, and providing high-accuracy decoding of natural videos from visual cortex activity in mice. These methods advance ethological neuroscience, enabling the study of naturalistic, high-dimensional behavior alongside neural dynamics.

Integrating behavior and neural data also reshapes experimental design. Real-time tracking enables closed-loop paradigms, in which specific postures or actions trigger neural stimulation or sensory feedback. For instance, optogenetic stimulation of the ventromedial hypothalamus in mice, combined with automated tracking of aggressive encounters, has revealed causal links between neural circuits and social behavior (Lin et al., 2011). Tools such as DeepLabStream (Schweihoff et al., 2021) and DeepLabCut-Live (Kane et al., 2020; Gonzalez et al., 2025) now make such experiments feasible with markerless, low-latency pose estimation. For instance, by integrating high resolution 3D tracking with location-triggered interactive rewards boxes and wireless neurophysiology in a closed loop setup, Nourizonoz et al. (2020) demonstrated the existence of hippocampal 3D place-cell-like activity in freely moving mouse lemurs.

Together, these advances are transforming ethological neuroscience. High-resolution behavioral tracking ensures that neural data are interpreted in the context of what animals are actually doing, while joint latent spaces and closed-loop designs reveal how internal brain states map onto overt behavior. ML thus provides the means to move from correlation to mechanism, linking the dynamics of the brain and body within naturalistic settings.

## 3 Discussion

### 3.1 Advances in ethological neuroscience

The rapid development of video-based tracking reflects a growing demand for precise behavioral quantification in ethological neuroscience. ML has advanced multiple new methods: supervised pose estimation and behavior classification enables detailed quantification of spontaneous behavior, social interactions and aggression, as well as closed loop experiments triggered by behavior sequences; unsupervised representation learning and variational embeddings reveal structured latent behavioral motifs directly from continuous behavior; and markerless 2D–3D tracking enables the study of gestures, group dynamics, and object manipulation in naturalistic environments. Together, these tools provide the spatial, temporal, and dimensional resolution needed to link neural activity with naturalistic, high-dimensional behavior. Importantly, these advances have enabled causal links between specific actions and neural circuits, detection of 3D place- and head-direction–tuned neurons, and decoding visual cortex activity, while scalable tracking in semi-natural arenas facilitates the study of collective behaviors. These developments align with calls for a computational neuroethology that unites brain and behavior (Datta et al., 2019).

### 3.2 Technical challenges

Despite the rapid adoption of ML, several methodological and infrastructural challenges remain. Behavioral data are inherently complex, requiring discretization into frames, trajectories, or annotated events. Most datasets rely on 2D video recordings, which obscure 3D movement, motivating the use of multi-view triangulation and 2D–3D reconstruction. Moreover, automated tracking produces high-dimensional datasets that require dimensionality reduction and complex analytical strategies to reveal latent structures relevant to neural dynamics.

Infrastructural demands are another bottleneck. High-resolution, multi-camera systems generate terabytes of data, which require robust storage, compression, and archiving. While computational resources such as GPUs have become more accessible, the greater challenge lies in standardization and reproducibility. Models trained in one lab often fail to generalize to new conditions, species, or lighting environments, underscoring the importance of consistent preprocessing pipelines and open-data practices.

Open-science provides a promising path forward. Sharing datasets, analysis pipelines, and model repositories accelerates method transfer and reproducibility while improving animal welfare through data reuse. Emerging resources such as standardized data formats, labeled datasets, and pretrained models, including the DLC ModelZoo and SuperAnimal models (Ye et al., 2024), promote transparency and cross-lab collaboration.

Finally, successful adoption of these techniques requires appropriate training. ML-based behavioral analysis depends on programming, data handling, and model interpretation skills that are not yet uniformly taught in neuroscience programs. Educational efforts such as Neuromatch Academy and the Cajal NeuroKit are helping democratize access, but broader institutional support and mentorship remain key to ensure that advances in ML benefit the field widely.

### 3.3 Future directions

The next phase of ethological neuroscience will likely be shaped by continued advances in ML and experimental design. Closed-loop paradigms that combine real-time tracking with neural manipulation are already allowing causal tests of circuit hypotheses in freely moving animals. At the same time, a gradual shift from strictly controlled laboratory conditions toward statistically controlled, naturalistic environments promises to improve ecological validity and reveal how the brain operates in complex, dynamic settings.

As ML methods mature, this synthesis between controlled experimentation and naturalistic observation could allow neuroscience to investigate brain–behavior relationships in the ecological contexts where they evolved. By integrating open-science practices and reproducible infrastructure, ethological neuroscience is poised to connect the precision of the laboratory with the richness of real-world behavior, offering a more complete understanding of how neural systems support adaptive action.

## 4 Conclusion

Machine learning has opened a new analytical era for behavioral neuroscience. It allows researchers to quantify the statistical structure and dynamics of behavior with a precision that parallels modern neural recording, enabling more direct links between brain activity and natural action. Beyond replacing manual scoring, these methods reveal the organization of behavior itself, exposing patterns that were previously invisible to human observers.

This transformation extends beyond technique. Treating behavior as a complex phenomenon invites a more integrative view of the nervous system, one that situates neural computation within the animal’s goals, body, and environment. Open-data, standardized pipelines, and shared pretrained models are already reinforcing this shift, making analyses more reproducible and comparable across species and contexts.

Ultimately, the convergence of behavioral measurement and computational modeling promises to move neuroscience toward a truly mechanistic understanding of how brains generate adaptive behavior. By embracing this perspective, ethological neuroscience can illuminate not just what the brain controls, but how it organizes action in the world.

## References

[B1] Abbaspoor S. Rahman K. Zinke W. Hoffman K. L. (2023). Learning of object-in-context sequences in freely-moving macaques. *bioRxiv [Preprint]* 10.1101/2023.12.11.571113 38168449 PMC10760043

[B2] Bain M. Nagrani A. Schofield D. Zisserman A. (2019). “Count, crop and recognise: fine-grained recognition in the wild,” in *Proceedings of the IEEE/CVF International Conference on Computer Vision Workshop*, (Montreal, BC), 10.48550/arXiv.1909.08950

[B3] Calhoun A. J. El Hady A. (2023). Everyone knows what behavior is but they just don’t agree on it. *Iscience* 26:108210. 10.1016/j.isci.2023.108210 37953955 PMC10638025

[B4] Calhoun A. J. Pillow J. W. Murthy M. (2019). Unsupervised identification of the internal states that shape natural behavior. *Nat. Neurosci.* 22 2040–2049. 10.1038/s41593-019-0533-x 31768056 PMC7819718

[B5] Charpentier M. J. Harté M. Poirotte C. de Bellefon J. M. Laubi B. Kappeler P. M. (2020). Same father, same face: deep learning reveals selection for signaling kinship in a wild primate. *Sci. Adv.* 6:eaba3274. 10.1126/sciadv.aba3274 32537486 PMC7253159

[B6] Couzin I. D. Heins C. (2023). Emerging technologies for behavioral research in changing environments. *Trends Ecol. Evol.* 38 346–354. 10.1016/j.tree.2022.11.008 36509561

[B7] Cunningham J. P. Yu B. M. (2014). Dimensionality reduction for large-scale neural recordings. *Nat. Neurosci.* 17 1500–1509. 10.1038/nn.3776 25151264 PMC4433019

[B8] Dankert H. Wang L. Hoopfer E. D. Anderson D. J. Perona P. (2009). Automated monitoring and analysis of social behavior in Drosophila. *Nat. Methods* 6 297–303. 10.1038/nmeth.1310 19270697 PMC2679418

[B9] Datta S. R. Anderson D. J. Branson K. Perona P. Leifer A. (2019). Computational neuroethology: a call to action. *Neuron* 104 11–24. 10.1016/j.neuron.2019.09.038 31600508 PMC6981239

[B10] Dunn T. W. Marshall J. D. Severson K. S. Aldarondo D. E. Hildebrand D. G. Chettih S. N. (2021). Geometric deep learning enables 3D kinematic profiling across species and environments. *Nat. Methods* 18 564–573. 10.1038/s41592-021-01106-6 33875887 PMC8530226

[B11] Ebrahimi A. S. Orlowska-Feuer P. Huang Q. Zippo A. G. Martial F. P. Petersen R. S. (2023). Three-dimensional unsupervised probabilistic pose reconstruction (3D-UPPER) for freely moving animals. *Sci. Rep.* 13:155. 10.1038/s41598-022-25087-4 36599877 PMC9813182

[B12] Gomez-Marin A. Paton J. Kampff A. Costa R. M. Mainen Z. F. (2014). Big behavioral data: psychology, ethology and the foundations of neuroscience. *Nat. Neurosci.* 17 1455–1462. 10.1038/nn.3812 25349912

[B13] Gonzalez M. Gradwell M. A. Thackray J. K. Temkar K. K. Patel K. R. Abraira V. E. (2025). Using DeepLabCut-Live to probe state dependent neural circuits of behavior with closed-loop optogenetic stimulation. *J. Neurosci. Methods* 110495. 10.1016/j.jneumeth.2025.110495 40436321

[B14] Gosztolai A. Günel S. Lobato-Ríos V. Pietro Abrate M. Morales D. Rhodin H. (2021). LiftPose3D, a deep learning-based approach for transforming two-dimensional to three-dimensional poses in laboratory animals. *Nat. Methods* 18 975–981. 10.1038/s41592-021-01226-z 34354294 PMC7611544

[B15] Graving J. M. Chae D. Naik H. Li L. Koger B. Costelloe B. R. (2019). DeepPoseKit, a software toolkit for fast and robust animal pose estimation using deep learning. *elife* 8:e47994. 10.7554/eLife.47994 31570119 PMC6897514

[B16] Grinciunaite A. Gudi A. Tasli E. Den Uyl M. (2016). “Human pose estimation in space and time using 3d cnn,” in *Proceedings of the European Conference on Computer Vision*, (Berlin), 10.1007/978-3-319-49409-8_5

[B17] Hirsch J. (1986). Nothing in neurobiology makes sense - except in the light of behaviour. *Contemp. Psychol.* 31 674–676. 10.1037/025029

[B18] Hsu A. I. Yttri E. A. (2021). B-SOiD, an open-source unsupervised algorithm for identification and fast prediction of behaviors. *Nat. Commun.* 12:5188. 10.1038/s41467-021-25420-x 34465784 PMC8408193

[B19] Kabra M. Robie A. A. Rivera-Alba M. Branson S. Branson K. (2013). JAABA: interactive machine learning for automatic annotation of animal behavior. *Nat. Methods* 10 64–67. 10.1038/nmeth.2281 23202433

[B20] Kane G. A. Lopes G. Saunders J. L. Mathis A. Mathis M. W. (2020). Real-time, low-latency closed-loop feedback using markerless posture tracking. *elife* 9:e61909. 10.7554/eLife.61909 33289631 PMC7781595

[B21] Karashchuk P. Rupp K. L. Dickinson E. S. Walling-Bell S. Sanders E. Azim E. (2021). Anipose: a toolkit for robust markerless 3D pose estimation. *Cell Rep.* 36:109730. 10.1016/j.celrep.2021.109730 34592148 PMC8498918

[B22] Kirkpatrick N. J. Butera R. J. Chang Y. H. (2022). DeepLabCut increases markerless tracking efficiency in X-ray video analysis of rodent locomotion. *J. Exp. Biol.* 225:jeb244540. 10.1242/jeb.244540 35950365

[B23] Krakauer J. W. Ghazanfar A. A. Gomez-Marin A. MacIver M. A. Poeppel D. (2017). Neuroscience needs behavior: correcting a reductionist bias. *Neuron* 93 480–490. 10.1016/j.neuron.2016.12.041 28182904

[B24] Krizhevsky A. Sutskever I. Hinton G. E. (2012). Imagenet classification with deep convolutional neural networks. *Adv. Neural Inform. Process. Syst.* 25 84–90. 10.1145/3065386

[B25] Lauer J. Zhou M. Ye S. Nath T. Feng G. Murthy V. (2022). Multi-animal pose estimation, identification and tracking with DeepLabCut. *Nature Methods* 19 496–504. 10.1038/s41592-022-01443-0 35414125 PMC9007739

[B26] Lin D. Boyle M. P. Dollar P. Lee H. Lein E. S. Perona P. (2011). Functional identification of an aggression locus in the mouse hypothalamus. *Nature* 470 221–226. 10.1038/nature09736 21307935 PMC3075820

[B27] Luxem K. Mocellin P. Fuhrmann F. Kürsch J. Miller S. R. Palop J. J. (2022). Identifying behavioral structure from deep variational embeddings of animal motion. *Commun. Biol.* 5:1267. 10.1038/s42003-022-04080-7 36400882 PMC9674640

[B28] Luxem K. Sun J. J. Bradley S. P. Krishnan K. Yttri E. Zimmermann J. (2023). Open-source tools for behavioral video analysis: Setup, methods, and best practices. *elife* 12:e79305. 10.7554/eLife.79305 36951911 PMC10036114

[B29] Mante V. Sussillo D. Shenoy K. V. Newsome W. T. (2013). Context-dependent computation by recurrent dynamics in prefrontal cortex. *Nature* 503 78–84. 10.1038/nature12742 24201281 PMC4121670

[B30] Mathis M. W. Mathis A. (2020). Deep learning tools for the measurement of animal behavior in neuroscience. *Curr. Opin. Neurobiol.* 60 1–11. 10.1016/j.conb.2019.10.008 31791006

[B31] Mlost J. Dawli R. Liu X. Costa A. R. Dorocic I. P. (2025). Evaluation of unsupervised learning algorithms for the classification of behavior from pose estimation data. *Patterns* 6:101237. 10.1016/j.patter.2025.101237 40486967 PMC12142628

[B32] Moll F. W. Würzler J. Nieder A. (2025). Learned precision tool use in carrion crows. *Curr. Biol.* 35 4845–4852. 10.1016/j.cub.2025.08.033 40934918

[B33] Monsees A. Voit K. M. Wallace D. J. Sawinski J. Charyasz E. Scheffler K. (2022). Estimation of skeletal kinematics in freely moving rodents. *Nat. Methods* 19, 1500–1509. 10.1038/s41592-022-01634-9 36253644 PMC9636019

[B34] Moore D. D. Walker J. D. MacLean J. N. Hatsopoulos N. G. (2022). Validating markerless pose estimation with 3D X-ray radiography. *J. Exp. Biol.* 225:jeb243998. 10.1242/jeb.243998 35466360 PMC9163444

[B35] Musall S. Kaufman M. T. Juavinett A. L. Gluf S. Churchland A. K. (2019). Single-trial neural dynamics are dominated by richly varied movements. *Nat. Neurosci.* 22 1677–1686. 10.1038/s41593-019-0502-4 31551604 PMC6768091

[B36] Nagy M. Naik H. Kano F. Carlson N. V. Koblitz J. C. Wikelski M. (2023). SMART-BARN: Scalable multimodal arena for real-time tracking behavior of animals in large numbers. *Sci. Adv.* 9:eadf8068. 10.1126/sciadv.adf8068 37656798 PMC10854427

[B37] Naik H. Chan A. H. H. Yang J. Delacoux M. Couzin I. D. Kano F. (2023). “3D-POP-An automated annotation approach to facilitate markerless 2D-3D tracking of freely moving birds with marker-based motion capture,” in *Proceedings of the IEEE/CVF Conference on Computer Vision and Pattern Recognition*, (Nashville, TN), 10.17617/3.HPBBC7

[B38] Nath T. Mathis A. Chen A. C. Patel A. Bethge M. Mathis M. W. (2019). Using DeepLabCut for 3D markerless pose estimation across species and behaviors. *Nat. Protocols* 14 2152–2176. 10.1038/s41596-019-0176-0 31227823

[B39] Nourizonoz A. Zimmermann R. Ho C. L. A. Pellat S. Ormen Y. Prévost-Solié C. (2020). EthoLoop: automated closed-loop neuroethology in naturalistic environments. *Nat. Methods* 17 1052–1059. 10.1038/s41592-020-0961-2 32994566

[B40] Pereira T. D. Shaevitz J. W. Murthy M. (2020). Quantifying behavior to understand the brain. *Nat. Neurosci.* 23 1537–1549. 10.1038/s41593-020-00734-z 33169033 PMC7780298

[B41] Pereira T. D. Tabris N. Matsliah A. Turner D. M. Li J. Ravindranath S. (2022). SLEAP: A deep learning system for multi-animal pose tracking. *Nat. Methods* 19 486–495. 10.1038/s41592-022-01426-1 35379947 PMC9007740

[B42] Pichler M. Hartig F. (2023). Machine learning and deep learning—A review for ecologists. *Methods Ecol. Evol.* 14 994–1016. 10.1111/2041-210X.14061

[B43] Reddy N. D. Guigues L. Pishchulin L. Eledath J. Narasimhan S. G. (2021). “Tessetrack: End-to-end learnable multi-person articulated 3d pose tracking,” in *Proceedings of the IEEE/CVF Conference on Computer Vision and Pattern Recognition*, (Vancouver, BC), 10.1109/CVPR46437.2021.01494

[B44] Redmon J. Divvala S. Girshick R. Farhadi A. (2016). “You only look once: Unified, real-time object detection,” in *Proceedings of the IEEE Conference on Computer Vision and Pattern Recognition*, (Seattle, WA), 10.1109/CVPR.2016.91

[B45] Romero-Ferrero F. Bergomi M. G. Hinz R. C. Heras F. J. H. de Polavieja G. G. (2019). idtracker.ai: tracking all individuals in small or large collectives of unmarked animals. *Nat. Methods* 16 179–182. 10.1038/s41592-018-0295-5 30643215

[B46] Saoud L. S. Sultan A. Elmezain M. Heshmat M. Seneviratne L. Hussain I. (2024). Beyond observation: Deep learning for animal behavior and ecological conservation. *Ecol. Informat.* 84:102893. 10.1016/j.ecoinf.2024.102893

[B47] Schneider S. Lee J. H. Mathis M. W. (2023). Learnable latent embeddings for joint behavioural and neural analysis. *Nature* 617 360–368. 10.1038/s41586-023-06031-6 37138088 PMC10172131

[B48] Schweihoff J. F. Loshakov M. Pavlova I. Kück L. Ewell L. A. Schwarz M. K. (2021). DeepLabStream enables closed-loop behavioral experiments using deep learningbased markerless, real-time posture detection. *Commun. Biol.* 4:130. 10.1038/s42003-021-01654-9 33514883 PMC7846585

[B49] Segalin C. Williams J. Karigo T. Hui M. Zelikowsky M. Sun J. J. (2021). The Mouse Action Recognition System (MARS) software pipeline for automated analysis of social behaviors in mice. *elife* 10:e63720. 10.7554/eLife.63720 34846301 PMC8631946

[B50] Sejnowski T. J. Churchland P. S. Movshon J. A. (2014). Putting big data to good use in neuroscience. *Nat. Neurosci.* 17 1440–1441. 10.1038/nn.3839 25349909 PMC4224030

[B51] Sheshadri S. Dann B. Hueser T. Scherberger H. (2020). 3D reconstruction toolbox for behavior tracked with multiple cameras. *J. Open Source Softw.* 5:1849. 10.21105/joss.01849

[B52] Vogg R. Lüddecke T. Henrich J. Dey S. Nuske M. Hassler V. (2025). Computer vision for primate behavior analysis in the wild. *Nat. Methods* 22 1154–1166. 10.1038/s41592-025-02653-y 40211003

[B53] von Ziegler L. M. Roessler F. K. Sturman O. Waag R. Privitera M. Duss S. N. (2024). Analysis of behavioral flow resolves latent phenotypes. *Nat. Methods* 21 2376–2387. 10.1038/s41592-024-02500-6 39533008 PMC11621029

[B54] Walter T. Couzin I. D. (2021). TRex, a fast multi-animal tracking system with markerless identification, and 2D estimation of posture and visual fields. *eLife* 10:e64000. 10.7554/eLife.64000 33634789 PMC8096434

[B55] Weinreb C. Pearl J. E. Lin S. Osman M. A. M. Zhang L. Annapragada S. (2024). Keypoint-MoSeq: parsing behavior by linking point tracking to pose dynamics. *Nat. Methods* 21 1329–1339. 10.1038/s41592-024-02318-2 38997595 PMC11245396

[B56] Wiltshire C. Lewis-Cheetham J. Komedová V. Matsuzawa T. Graham K. E. Hobaiter C. (2023). DeepWild: application of the pose estimation tool DeepLabCut for behaviour tracking in wild chimpanzees and bonobos. *J. Anim. Ecol.* 92 1560–1574. 10.1111/1365-2656.13932 37165474

[B57] Wittek N. Wittek K. Keibel C. Güntürkün O. (2023). Supervised machine learning aided behavior classification in pigeons. *Behav. Res. Methods* 55 1624–1640. 10.3758/s13428-022-01881-w 35701721 PMC10250476

[B58] Yamins D. L. DiCarlo J. J. (2016). Using goal-driven deep learning models to understand sensory cortex. *Nat. Neurosci.* 19 356–365. 10.1038/nn.4244 26906502

[B59] Ye S. Filippova A. Lauer J. Schneider S. Vidal M. Qiu T. (2024). SuperAnimal pretrained pose estimation models for behavioral analysis. *Nat. Commun.* 15:5165. 10.1038/s41467-024-48792-2 38906853 PMC11192880

